# The Relationship Between Nomophobia and Maladaptive Coping Styles in a Sample of Italian Young Adults: Insights and Implications From a Cross-Sectional Study

**DOI:** 10.2196/13154

**Published:** 2019-04-24

**Authors:** Nicola Luigi Bragazzi, Tania Simona Re, Riccardo Zerbetto

**Affiliations:** 1 Department of Health Sciences University of Genoa Genova Italy; 2 United Nations Educational, Scientific, and Cultural Organization Chair Genoa Italy; 3 Gestalt Study Center Milan Italy

**Keywords:** nomophobia, coping styles, behavioral addiction, mobile phone addiction

## Abstract

**Background:**

Information technologies have become an integral part of the modern society; however, it is speculated that their overuse would result in addiction. Nomophobia refers to the irrational fear of being out of contact with virtual communication platforms. Generally, upon exposure to stress, humans adjust by employing cognitive mechanisms and behavioral efforts known as coping strategies.

**Objective:**

The goal of the research was to explore coping styles implemented in subjects with nomophobia.

**Methods:**

This was a cross-sectional study involving young adult participants (undergraduate students and younger subjects) who were recruited via an online survey using a snowball approach. The Italian version of the Nomophobia Questionnaire was administered to subjects. The measurement of coping styles was done using the 28-item Brief COPE questionnaire. Continuous data were computed as means and standard deviations, whereas categorical data were expressed as percentages, where appropriate. Correlation analysis was performed between the Nomophobia Questionnaire and Brief COPE scores. Multivariate regression analyses were conducted in order to shed light on the determinants of each coping style and its association with nomophobia.

**Results:**

A total of 403 subjects took part in the study. Subjects with higher nomophobia scores responded when confronted with stress with behavioral disengagement (*r*=.16, *P*<.001), denial (*r*=.19, *P*<.001), self-blame (*r*=.12, *P*=.02), self-distraction (*r*=.22, *P*<.001), venting (*r*=.28, *P*<.001), use of emotional (*r*=.25, *P*<.001), and instrumental support (*r*=.16, *P*=.001).

**Conclusion:**

Nomophobia subjects adopt maladaptive coping strategies when confronted with stress. The acknowledgment of how nomophobia subjects react provides insight and introduces a focus for preventative and interventional measures in this population.

## Introduction

In the digital era, the advent of new information and communication technologies (ICTs) has provided an avenue for rapid communication, efficient data retrieval, and access to the internet, the widest world global communication network [1]. The pervasive and ubiquitous use of ICTs has raised the possibility of whether their overuse/misuse can eventually result in addiction [2]. Portable phones have become an indispensable tool of our daily life [3], and preoccupation with up-to-the-minute mobile apps can indeed foster an environment where people tend to spend more time with technology than with their peers [4]. Nomophobia, a portmanteau of the words “no mobile phone” and “phobia,” represents a new emerging psychological construct describing the discomfort of being without mobile contact and the irrational fear and anxiety arising from the feeling of disconnection from virtual communication platforms [5,6].

Inspecting the construct from a broader aspect, various elements have been suggested to play an integral part of nomophobia. Of note, ringxiety (combination of the words “ring” and ”anxiety”) is the state of disquietude and malaise, characterized by the need to constantly check the mobile phone to see whether messages or calls have been received. Additional features include (1) incurring debts as a result of over-expenditure on mobile technology, (2) the need to sleep with phone in close proximity, which may lead to impaired, fragmented sleep, and (3) the unease of turning the phone off [7].

Prevalence of nomophobia is highly variable, ranging from approximately 20% to 70%, depending on the study design, country, population, sampling technique, and cultural habits [7]. However, a body of circumstantial evidence seems to suggest that it is quite widespread and common.

In response to daily life stressors, humans can adopt coping strategies in order to deal with them. Coping refers to any adjustment mechanism or skill, whether behavioral or cognitive, that mitigates or counteracts a perceived psychological stress or event [8,9]. The current paradigm classifies coping styles into two broad categories: problem-focused and emotional-focused. The former target causes of stress aiming at achieving stress reduction or removal, whereas the latter try to diminish the negative emotional response associated with stressors [10].

In view of the ever-increasing attachment to ICTs, the emerging fear of being disconnected and the resultant deleterious impact on daily functioning and psychological well-being, this study investigated coping skills implemented in subjects with nomophobia.

## Methods

### Study Design and Participant Selection

For this cross-sectional study, participants (mainly undergraduate students and younger subjects) were recruited via an online survey using a snowball approach. For the purpose, Google Forms, an open-source tool for developing online questionnaires, was used.

All procedures were carried out according to the ethical standards of the 1964 Helsinki declaration and its subsequent amendments. The study protocol NOMO-001234-016 was reviewed and approved by the United Nations Educational, Scientific, and Cultural Organization Chair, “Health Anthropology Biosphere and Healing Systems,” University of Genoa, Genoa, Italy. Participants signed a written informed consent and were advised of the aims of the survey and its anonymous nature, in that data could not be associated with the single individual who had provided them and were analyzed on an aggregated basis.

### Instruments

The Italian version of the Nomophobia Questionnaire (NMP-Q), translated from the instrument originally developed by Yildirim and Correia [11], was administered. Exploratory factor analysis had previously demonstrated good psychometric properties of the instrument (Cronbach alpha coefficient of .95, .94, .89, and .88 for the overall questionnaire and its three factors, respectively). Furthermore, validity of the questionnaire was confirmed by conducting regression analysis with number of hours spent on mobile phone as regressor. However, in the Italian version a three-factor structure was found (factor 1: not being able to access information, factor 2: giving up convenience/losing connectedness, and factor 3: not being able to communicate), which is different from the other translated versions (in Spanish, Persian, or Chinese) [12-15].

Coping styles, the strategies exploited by individuals to cope with problems and stress, were investigated by administering the 28-item Brief COPE questionnaire, originally developed by Carver [16], based on Carver and Scheier’s self-regulation theory. This instrument was translated into the Italian language by Conti [17] and demonstrated good reliability and internal consistency. The questionnaire comprises 14 subscales: self-distraction, active coping, denial, substance use, use of emotional support, use of instrumental support, behavioral disengagement, venting, positive reframing, planning, humor, acceptance, religion, and self-blame. The score for each item of the instrument ranges on a Likert scale from 1 (not at all) to 4 (a lot) and, given that the score of a subscale can be obtained by summing two items, its score ranges from 2 to 8. Moreover, besides computing the score of each subscale, overall coping strategy was defined as problem-oriented if the sum of the problem-solving items of the questionnaire (items 2, 7, 10, 14, 23, and 25) was greater than the sum of the emotional items (5, 12, 15, 17, 22, and 27). When the opposite was true, the coping style was defined as emotion-oriented. Coping styles were also grouped into adaptive or maladaptive strategies and analyzed accordingly.

### Statistical Analysis

Continuous data were computed as means and standard deviations, whereas categorical data were expressed as percentages, where appropriate.

Correlation analysis was performed between the NMP-Q and Brief COPE scores. The magnitude of the Pearson coefficient was interpreted using the following rule of thumb developed by Hinkle and coauthors [18]: the strength of the correlation was deemed negligible if the *r* coefficient ranged from 0 to .30, low from .30 to .50, moderate from .50 to .70, high from .70 to .90, and very high from .90 to 1.00. Correlation was also computed between the Brief COPE scores and the number of hours spent on mobile phones using the Spearman rank coefficient.

Furthermore, multivariate regression analyses were conducted in order to shed light on the determinants of each coping style. All statistical analyses were performed using the commercial software SPSS Statistics version 21.0.0 (IBM Corp). Figures with *P* values less than .05 were considered statistically significant.

## Results

A total of 403 subjects aged 27.91 (SD 8.63) years (males 160/403, 39.7%, and females 243/403, 60.3%) took part in the study. In detail, 11.2% (45/403) of subjects spent less than 1 hour on their mobile phone per day, 23.3% (94/403) spent 1 to 2 hours, 17.1% (69/403) spent 2 to 3 hours, 14.4% (58/403) spent 3 to 4 hours, 11.9% (48/403) spent 4 to 5 hours, 7.2% (29/403) spent 5 to 7 hours, 8.9% (36/403) spent 7 to 9 hours, and 6.0% (24/403) spent more than 10 hours. Stratifying the population according to the severity of the psychological construct, 51.1% (206/403) of individuals suffered from mild nomophobia, whereas 41.4% (167/403) and 7.4% (30/403) individuals reported moderate and severe nomophobia, respectively. For further details related to the characteristics of the sample, the reader is referred to our previous publication [7]. Brief COPE scores are reported in Table 1, while Table 2 lists the correlations between these scores and the scores obtained with the NMP-Q instrument.

Behavioral disengagement correlated with the overall score (*P*=.001) and with D2 (*P*<.001), whereas denial correlated with overall score (*P*<.001), D1 (*P*=.02), D2 (*P*<.001), and D3 (*P*=.03). Religion correlated with D2 (*P*=.04), self-blame was associated with overall score (*P*=.02), D1 (*P*=.006), and D3 (*P*=.02). Self-distraction correlated with overall score (*P*<.001), D1 (*P*<.001), D2 (*P*=.002), and D3 (*P*<.001). Use of emotional support was associated with overall score (*P*<.001), D1 (*P*<.001), D2 (*P*<.001), and D3 (*P*<.001). Use of instrumental support correlated with overall score (*P*=.001), D1 (*P*=.003), D2 (*P*=.02), and D3 (*P*=.002), while venting was associated with overall score (*P*<.001), D1 (*P*<.001), D2 (*P*<.001), and D3 (*P*<.001). Planning negatively correlated with D2 (*P*=.04). Denial (*P*=.004), self-blame (*P*=.004), self-distraction (*P*<.001), use of emotional support (*P*=.003), use of instrumental support (*P*=.003), and venting (*P*=.006) correlated with the number of hours spent on mobile phones (Table 3).

At the multivariate regression analysis, several predictors were found for adaptive coping strategies scores (Table 4). In particular, active coping was associated with D2 (*P*=.004), whereas planning with D1 (*P*=.01) and D2 (*P*=.002). Positive reframing correlated with D3 (*P*=.04), while religion correlated with D1 (*P*<.001) and D2 (*P*=.002).

Concerning maladaptive coping strategies (Table 5), behavioral disengagement and denial correlated with D2 (*P*<.001 for both). Substance use was found to be associated with D1 (*P*=.01) and D2 (*P*=.002), while venting with D1 (*P*=.04).

**Table 1:** Scores of the Brief COPE questionnaire.

Subscale	Score, mean (SD)
Acceptance	5.9 (1.3)
Active coping	6.5 (1.5)
Behavioral disengagement	3.2 (1.4)
Denial	3.1 (1.4)
Humor	4.4 (1.6)
Planning	6.5 (1.4)
Positive reframing	5.6 (1.6)
Religion	4.0 (2.2)
Self-blame	5.8 (1.4)
Self-distraction	5.2 (1.6)
Substance use	2.5 (1.2)
Use of emotional support	5.2 (1.8)
Use of instrumental support	5.7 (1.6)
Venting	5.0 (1.6)

**Table 2:** Correlation between Brief COPE and Nomophobia Questionnaire scores.

Subscale	Overall score		D1^a^ (not being able to access information)		D2^b^ (giving up convenience/losing connectedness)		D3^c^ (not being able to communicate)	
	*r*^d^	*P* value	*r*	*P* value	*r*	*P* value	*r*	*P* value
Acceptance	–.07	.14	–.03	.54	–.09	.09	–.07	.15
Active coping	.02	.76	.06	.25	–.08	.12	.07	.18
Behavioral disengagement	.16	.001	.09	.06	.25	<.001	.08	.11
Denial	.19	<.001	.12	.02	.26	<.001	.12	.03
Humor	.07	.19	.06	.25	.09	.08	.03	.56
Planning	–.02	.69	.05	.33	–.11	.04	.01	.81
Positive reframing	–.003	.96	–.02	.70	–.04	.47	.05	.35
Religion	.01	.71	–.09	.07	.10	.04	.02	.63
Self-blame	.12	.02	.14	.006	.06	.22	.11	.02
Self-distraction	.22	<.001	.22	<.001	.16	.002	.20	<.001
Substance use	–.001	.98	–.06	.20	.09	.09	–.03	.49
Use of emotional support	.25	<.001	.23	<.001	.20	.001	.22	<.001
Use of instrumental support	.16	.001	.15	.003	.11	.02	.16	.002
Venting	.28	<.001	.27	<.001	.22	<.001	.24	<.001

^a^D1: first dimension (not being able to access information).

^b^D2: second dimension (giving up convenience/losing connectedness).

^c^D3: third dimension (not being able to communicate).

^d^*r*: Pearson coefficient.

**Table 3:** Correlation between Brief COPE scores and number of hours spent on mobile phones.

Subscale	Spearman correlation with number of hours	
	rho	*P* value
Acceptance	–.02	.74
Active coping	–.08	.09
Behavioral disengagement	.05	.30
Denial	.14	.004
Humor	–.04	.47
Planning	–.08	.10
Positive reframing	–.04	.44
Religion	–.03	.50
Self-blame	.14	.004
Self-distraction	.20	<.001
Substance use	–.03	.58
Use of emotional support	.15	.003
Use of instrumental support	.15	.003
Venting	.14	.006

**Table 4:** Multivariate regression of adaptive coping strategies scores and their association with nomophobia.

Parameter		Nonstandardized coefficients		Standardized coefficient (beta^b^)	T value	*P* value
		B^a^	SD			
**Acceptance**						
	(Constant)	6.28	.46	—^c^	13.78	<.001
	Number of hours spent on a mobile phone	.002	.04	.003	0.05	.96
	Age	–.01	.01	–.05	–0.87	.38
	Gender	–.14	.14	–.05	–0.99	.32
	Schooling level	.06	.08	.04	0.72	.47
	D1^d^ (not being able to access information)	.01	.01	.07	0.92	.36
	D2^e^ (giving up convenience/losing connectedness)	–.01	.01	–.10	–1.35	.18
	D3^f^ (not being able to communicate)	–.01	.01	–.05	–0.74	.46
**Active coping**						
	(Constant)	5.40	.50	—	10.82	<.001
	Number of hours spent on a mobile phone	–.02	.04	–.03	–0.57	.57
	Age	.01	.01	.04	0.78	.44
	Gender	–.09	.15	–.03	–0.55	.58
	Schooling level	.22	.09	.13	2.48	.014
	D1 (not being able to access information)	.02	.01	.12	1.61	.11
	D2 (giving up convenience/losing connectedness)	–.03	.01	–.21	–2.91	.004
	D3 (not being able to communicate)	.02	.01	.13	1.81	.07
**Humor**						
	(Constant)	4.70	.54	—	8.77	<.001
	Number of hours spent on a mobile phone	–.05	.05	–.06	–1.00	.32
	Age	–.01	.01	–.05	–0.86	.39
	Gender	–.37	.17	–.12	–2.25	.025
	Schooling level	.10	.10	.06	1.05	.30
	D1 (not being able to access information)	.01	.02	.04	0.45	.65
	D2 (giving up convenience/losing connectedness)	.02	.01	.12	1.62	.11
	D3 (not being able to communicate)	–.01	.01	–.03	–0.44	.66
**Planning**						
	(Constant)	6.14	.48	—	12.68	<.001
	Number of hours spent on a mobile phone	–.05	.04	–.07	–1.20	.23
	Age	–.002	.01	–.01	–0.24	.81
	Gender	–.28	.15	–.10	–1.86	.064
	Schooling level	.21	.09	.12	2.40	.02
	D1 (not being able to access information)	.03	.01	.19	2.53	.012
	D2 (giving up convenience/losing connectedness)	–.03	.01	–.22	–3.10	.002
	D3 (not being able to communicate)	.01	.01	.05	0.74	.46
**Positive reframing**						
	(Constant)	6.06	.54	—	11.18	<.001
	Number of hours spent on a mobile phone	–.01	.05	–.02	–0.28	.78
	Age	–.004	.01	–.02	–0.39	.69
	Gender	–.15	.17	–.05	–0.91	.37
	Schooling level	–.02	.10	–.01	–0.22	.82
	D1 (not being able to access information)	–.01	.02	–.05	–0.67	.50
	D2 (giving up convenience/losing connectedness)	–.01	.01	–.09	–1.19	.24
	D3 (not being able to communicate)	.03	.01	.15	2.03	.04
**Religion**						
	(Constant)	8.10	.72	—	11.32	<.001
	Number of hours spent on a mobile phone	–.05	.06	–.05	–0.88	.38
	Age	–.02	.01	–.09	–1.73	.09
	Gender	–.74	.22	–.17	–3.36	.001
	Schooling level	–.56	.13	–.21	–4.33	<.001
	D1 (not being able to access information)	–.07	.02	–.26	–3.62	<.001
	D2 (giving up convenience/losing connectedness)	.05	.02	.21	3.15	.002
	D3 (not being able to communicate)	.03	.02	.11	1.59	.11
**Use of emotional support**						
	(Constant)	2.37	.57	—	4.16	<.001
	Number of hours spent on a mobile phone	.01	.05	.01	0.11	.92
	Age	–.02	.01	–.08	–1.61	.11
	Gender	.78	.18	.22	4.44	<.001
	Schooling level	.32	.10	.15	3.10	.002
	D1 (not being able to access information)	.02	.02	.07	1.00	.32
	D2 (giving up convenience/losing connectedness)	.02	.01	.09	1.25	.21
	D3 (not being able to communicate)	.01	.01	.07	0.99	.33
**Use of instrumental support**						
	(Constant)	4.51	.54	—	8.31	<.001
	Number of hours spent on a mobile phone	.04	.05	.05	0.76	.45
	Age	–.02	.01	–.09	–1.70	.09
	Gender	.35	.17	.11	2.09	.04
	Schooling level	.12	.10	.06	1.20	.23
	D1 (not being able to access information)	.01	.015	.04	0.47	.64
	D2 (giving up convenience/losing connectedness)	–.001	.01	–.01	–0.08	.94
	D3 (not being able to communicate)	.02	.01	.10	1.38	.17

^a^B: nonstandardized regression coefficient.

^b^Beta: standardized regression coefficient.

^c^Not applicable.

^d^D1: first dimension (not being able to access information).

^e^D2: second dimension (giving up convenience/losing connectedness).

^f^D3: third dimension (not being able to communicate).

**Table 5:** Multivariate regression of maladaptive coping strategies scores and their association with nomophobia.

Parameter		Nonstandardized coefficients		Standardized coefficient (beta^b^)	T value	*P* value
		B^a^	SD			
**Behavioral disengagement**						
	(Constant)	4.37	.46	—^c^	9.47	<.001
	Number of hours spent on a mobile phone	–.04	.04	–.05	–0.92	.36
	Age	–.02	.01	–.10	–1.95	.05
	Gender	–.24	.14	–.08	–1.68	.09
	Schooling level	–.19	.08	–.11	–2.26	.03
	D1^d^ (not being able to access information)	–.01	.01	–.05	–0.67	.50
	D2^e^ (giving up convenience/losing connectedness)	.05	.01	.33	4.69	<.001
	D3^f^ (not being able to communicate)	–.01	.01	–.07	–0.94	.35
**Denial**						
	(Constant)	3.37	.46	—	7.35	<.001
	Number of hours spent on a mobile phone	.03	.04	.04	0.74	.46
	Age	.000	.01	–.001	–0.02	.98
	Gender	.12	.14	.04	0.83	.41
	Schooling level	–.29	.08	–.17	–3.47	.001
	D1 (not being able to access information)	–.01	.01	–.05	–0.72	.47
	D2 (giving up convenience/losing connectedness)	.05	.01	.30	4.40	<.001
	D3 (not being able to communicate)	–.01	.01	–.07	–0.94	.35
**Self–blame**						
	(Constant)	4.92	.48	—	10.21	<.001
	Number of hours spent on a mobile phone	.04	.04	.06	0.94	.35
	Age	–.01	.01	–.07	–1.18	.24
	Gender	.13	.15	.05	0.89	.37
	Schooling level	.13	.09	.08	1.45	.15
	D1 (not being able to access information)	.02	.01	.10	1.29	.20
	D2 (giving up convenience/losing connectedness)	–.01	.01	–.07	–1.01	.31
	D3 (not being able to communicate)	.01	.01	.06	0.89	.38
**Self-distraction**						
	(Constant)	3.93	.52	—	7.54	<.001
	Number of hours spent on a mobile phone	.07	.04	.09	1.60	.11
	Age	–.01	.01	–.06	–1.07	.29
	Gender	.38	.16	.12	2.38	.02
	Schooling level	–.02	.09	–.01	–0.18	.86
	D1 (not being able to access information)	.02	.01	.11	1.49	.14
	D2 (giving up convenience/losing connectedness)	–.01	.01	–.03	–0.45	.65
	D3 (not being able to communicate)	.02	.01	.09	1.28	.20
**Substance abuse**						
	(Constant)	2.39	.39	—	6.08	<.001
	Number of hours spent on a mobile phone	.05	.03	.09	1.51	.13
	Age	.01	.01	.06	1.17	.24
	Gender	–.27	.12	–.11	–2.21	.03
	Schooling level	.07	.07	.05	1.01	.32
	D1 (not being able to access information)	–.03	.01	–.20	–2.56	.01
	D2 (giving up convenience/losing connectedness)	.03	.01	.23	3.18	.002
	D3 (not being able to communicate)	–.004	.01	–.04	–0.50	.62
**Venting**						
	(Constant)	2.15	.52	—	4.13	<.001
	Number of hours spent on a mobile phone	.02	.04	.02	0.40	.69
	Age	.01	.01	.07	1.23	.22
	Gender	.66	.16	.20	4.09	<.001
	Schooling level	.09	.09	.05	0.93	.36
	D1 (not being able to access information)	.03	.01	.15	2.04	.04
	D2 (giving up convenience/losing connectedness)	.01	.01	.07	1.06	.29
	D3 (not being able to communicate)	.01	.01	.06	0.80	.43

^a^B: nonstandardized regression coefficient.

^b^Beta: standardized regression coefficient.

^c^Not applicable.

^d^D1: first dimension (not being able to access information).

^e^D2: second dimension (giving up convenience/losing connectedness).

^f^D3: third dimension (not being able to communicate).

## Discussion

### Principal Findings

In this study, we found that when confronted with stress, subjects with higher nomophobia scores were significantly more likely to respond with behavioral disengagement, denial, self-blame, self-distraction, venting, and use of emotional and instrumental support. Similarly, increased number of hours spent on mobile phones correlated with significantly higher use of denial, self-blame, self-distraction, venting, and use of emotional and instrumental support. Taken together, we found that nomophobic subjects tended to adopt dysfunctional coping strategies, which has been revealed to be independently associated with anxiety [19,20].

Our findings are consistent with the results published by Dziurzyńska et al [21], demonstrating an increased likelihood of individuals at risk of mobile phone addiction to cope with stress using substitute gratification, resignation, passivity, dejection, blaming others, pitying themselves, and hopelessness. Furthermore, nomophobic subjects were shown to ruminate over their suffering, withdraw from social interactions, and react with aggressiveness.

Roberts and collaborators [22] disclosed that heavy use of cellular communication during stress is regarded as a form of self-distraction or substitute gratification, or a kind of addiction. Moreover, subjects with neuroticism reported using their mobile phone and internet to feel a sense of belonging and escape loneliness as a means to cope with stress [23].

Li et al [24] inquired into problematic internet use and its relationship to stressful life events and coping styles. Subjects’ reported high preoccupation with the internet correlated positively with self-blame, fantasy, and withdrawal scores. Additionally, higher internet addiction scores correlated with social-communication and daily hassle scores. Further, stressful life events and generalized problematic internet use were shown to be mediated by avoidant coping style. Similarly, chronic stress, low emotional stability, female gender, and young age were significantly associated with excessive and dysfunctional mobile use [25]. Among other possibilities on mobile phones are game playing and music listening, which have been consistently shown to provide means to respond to stress by diverting attention from problems by seeking substitute gratification [4]. Similarly, subjects with pathological use of internet games and addiction to computer games employed nonadaptive coping [26,27]. Wan et al [28] indicated that addiction to online game playing provided means of emotional coping with stress allowing subjects to escape loneliness and isolation and relieve anger and frustration.

Arpaci et al [29] explored the effect of mindfulness on the relationship between attachment styles and nomophobia. Their analysis demonstrated a positive direct effect of avoidant and anxious attachment styles on nomophobia. Emotional and dependent people display higher stress when they have no access to their phones. Thus their anxious attachment is projected upon an object, in that case a mobile phone. Komorowska-Pudlo [30] found that anxious attachment correlated with denial, venting, self-blame, and suppression of activities. Further, subjects with avoidant attachment style used less active coping mechanisms, preferring suppression of activities.

Socioeconomic factors such as education have been shown to have substantial influence on coping mechanisms. Roohafza et al [31] demonstrated a positive correlation between higher education levels and adaptive coping strategies, and an inverse relationship to maladaptive coping styles was found. Similarly, low education and low income were linked to maladaptive coping strategies attainment.

In the study conducted by Matud et al [32], women scored significantly higher in emotional and avoidance coping styles as compared to men. Counterintuitively, in our study, male gender in nomophobic subjects was a predictor for avoidant coping style, self-distraction, venting, and use of emotional support. Dissimilarly, turning to religion, sense of humor, and planning were adopted by female nomophobic subjects.

### Limitations

This study has some limitations that warrant discussion. The major shortcoming is given by the study design: being cross-sectional and not longitudinal, our investigation cannot capture dynamic relationships between the variables under scrutiny. Second, like other subjective self-reported studies, it is not immune from the response bias. Furthermore, the Brief COPE measure assumes general tendency toward a specific strategy rather than opting for a dynamic approach in dealing with encountered problems. Our study has some strengths including its novelty and the use of a relatively large number of subjects, who were, however, selected using a nonprobability purposive sampling technique.

From a clinical standpoint, this study has practical implications: the acknowledgment of how nomophobic subjects approach and cope with stress can potentially provide information that informs the design of ad hoc preventative and interventional measures for this particular population. The major focus of these interventions should be to foster a deeper awareness of the deleterious psychological impact of nomophobia and the problematic use of one’s own mobile phone on daily life and social activities. The tools, being validated, psychometrically sound, and reliable, can be used to measure cognitive and behavioral changes, monitoring the effectiveness of the interventions.

### Conclusion

The findings of this study suggest a major adoption of maladaptive coping strategies in nomophobic subjects. Different predictors of the association between nomophobia and coping styles were found, including gender, number of hours spent on mobile devices, and socioeconomic status. Among nomophobic subjects with higher schooling levels, positive adaptive coping strategies including active coping, planning, and use of emotional support were documented. In contrast, lower schooling level was a predictor for maladaptive coping strategies such as behavioral disengagement and denial. These results advance the burgeoning field of cyberpsychology and offer insights for the development and implementation of preventive strategies. Further high-quality studies, especially longitudinal ones and randomized controlled trials, are needed to confirm and replicate our findings.

## Abbreviations

ICT: information and communication technology
NMP-Q: Nomophobia Questionnaire
